# Sequence Variants of *ADIPOQ* and Association with Type 2 Diabetes Mellitus in Taiwan Chinese Han Population

**DOI:** 10.1155/2014/650393

**Published:** 2014-07-10

**Authors:** Ming-Kai Tsai, Hui-Min David Wang, Jeng-Chuan Shiang, I-Hung Chen, Chih-Chiang Wang, Ya-Fen Shiao, Wen-Sheng Liu, Tai-Jung Lin, Tsung-Ming Chen, Ya-Huey Chen

**Affiliations:** ^1^Division of Nephrology, Department of Medicine, Kaohsiung Armed Forces General Hospital, Kaohsiung 80284, Taiwan; ^2^Department of Fragrance and Cosmetic Science, Kaohsiung Medical University, Kaohsiung 80708, Taiwan; ^3^Graduate Institute of Natural Products, Kaohsiung Medical University, Kaohsiung 80708, Taiwan; ^4^Asia-Pacific Biotech Developing, Inc., Kaohsiung 80681, Taiwan; ^5^Department of Pharmacy and Graduate Institute of Pharmaceutical Technology, Tajen University, Pingtung 90741, Taiwan; ^6^Department of Physiology, College of Medicine, National Cheng Kung University, 1 University Road, Tainan 70101, Taiwan; ^7^Department and Graduate Institute of Aquaculture, National Kaohsiung Marine University, Kaohsiung 81157, Taiwan; ^8^Center for Molecular Medicine, China Medical University Hospital, Taichung 40477, Taiwan; ^9^Graduate Institute of Cancer Biology, China Medical University, Cancer Center Building 8F, No. 6 Hsueh-Shih Road, Taichung 40477, Taiwan

## Abstract

Diabetes is a serious global health problem. Large-scale genome-wide association studies identified loci for type 2 diabetes mellitus (T2DM), including adiponectin (*ADIPOQ*) gene and transcription factor 7-like 2 (*TCF7L2*), but few studies clarified the effect of genetic polymorphisms of *ADIPOQ* and *TCF7L2* on risk of T2DM. We attempted to elucidate association between T2DM and polymorphic variations of both in Taiwan's Chinese Han population, with our retrospective case-control study genotyping single nucleotide polymorphisms (SNPs) in *ADIPOQ* and *TCF7L2* genes both in 149 T2DM patients and in 139 healthy controls from Taiwan. Statistical analysis gauged association of these polymorphisms with risk of T2DM to show *ADIPOQ* rs1501299 polymorphism variations strongly correlated with T2DM risk (*P* = 0.042), with rs2241766 polymorphism being not associated with T2DM (*P* = 0.967). However, both polymorphisms rs7903146 and rs12255372 of *TCF7L2* were rarely detected in Taiwanese people. This study avers that *ADIPOQ* rs1501299 polymorphism contributes to risk of T2DM in the Taiwanese population.

## 1. Introduction

Global prevalence of type 2 diabetes mellitus (T2DM) is 6% but is expected to rise due to aging population [1]; 2012 saw some 1.6 million patients (~7% of total population) in Taiwan suffering from it, with ~90% of these cases diagnosed as T2DM, non-insulin-dependent diabetes characterized by impaired insulin secretion in peripheral tissues. It accounts for up to 11.5% of healthcare costs in Taiwan annually. Factors such as age, weight, diet, lifestyle, and family history are linked therewith [1]; higher concordance rate among monozygotic versus dizygotic twins and sibling risk manifested itself, and prevalence differs among racial groups [2], suggesting genetic contribution to risk of T2DM.

Genome-wide studies identify diabetes susceptibility loci on chromosomes 3q27 and 10q25.3, where adiponectin genes (*ADIPOQ* or* APM1*) and* TCF7L2* are located, respectively [3]. Human adiponectin, encoded by* ADIPOQ* gene, is an adipose-specific secretory protein involved in many metabolic processes: for example, glucose modulation and fatty acid oxidation [4]. Adiponectin level positively correlates with weight [5], T2DM [5], insulin resistance [6], and cardiovascular diseases [5, 7] but negatively with insulin level [8]. Polymorphisms of* ADIPOQ* are linked with metabolic syndromes: for example, insulin resistance, abdominal obesity, impaired glucose tolerance, dyslipidemia, hypertension, increased fasting glucose [9, 10], and plasma adiponectin level [11, 12]. Also, haplotype analysis of* ADIPOQ* showed significant association with obesity and insulin resistance [13, 14]. Two* ADIPOQ* SNPs, rs2241766 and rs1501299, showed significant association with risk of T2DM in the Japanese population [15, 16], yet this phenomenon did not appear in the French population [17]. Interestingly, persons without family history of diabetes revealed stark correlation of rs2241766 SNP, obesity, and insulin sensitivity among German people [18], making the role of* ADIPOQ* gene in T2DM controversial. Any relation between these SNPs and T2DM in the Taiwanese remains unclear. Human transcription factor 7-like 2 (*TCF7L2*; a.k.a. TCF4) [19] is involved in beta-catenin-dependent Wnt signaling pathway, playing roles in pancreatic and intestinal endocrine cells [20, 21]. Polymorphism of* TCF7L2* gene correlates with T2DM risk and progression in multiple ethnicities [22–26]. Strongly associated variants rs12255372 and rs7903146 were cited as risk factors [27], while information about* TCF7L2* and* ADIPOQ* on T2DM risk is scant in Taiwanese populations; we researched both genes.

## 2. Materials and Methods

### 2.1. Sample Collection

We recruited 149 individuals with T2DM and 139 healthy controls, as diagnosed by Dr. Ming-Kai Tsai, with basic characteristics of both groups summarized in Table 1; 3 mL blood samples with EDTA anticoagulants were collected from Kaohsiung Armed Forces General Hospital with informed consent. Genomic DNA was isolated from blood samples using the DNA isolation kit (QIAamp DNA Blood Mini Kit, Qiagen, Valencia, Germany).

### 2.2. Marker Selection and Genotyping of SNPs

In* ADIPOQ* and* TCFL2*, 33 and 84 SNPs were reported, respectively, from dbSNP database (http://www.ncbi.nlm.nih.gov/SNP). SNPs were based on genetic information for the Chinese (CHB) and the Japanese (JPT) in HapMap (www.hapmap.org). Allele-specific polymerase chain reaction (AS-PCR) analysis was performed with allele-specific primer sets (Table 2) on genotype alleles of* ADIPOQ* (rs2241766 and rs1501299) and* TCF7L2* (rs7903146 and rs12255372). In brief, forward primers specifically complementary to each SNP allele were designed and two PCR reactions were conducted in parallel to determine the allele. PCR used MyCycle thermal cycler (USA) containing 1.25 U of Taq DNA polymerase (TaKaRa), 1X PCR buffer, 200 umol/L dNTP, 100 ng of each primer, and 10 ng of genomic DNA. Amplification was through initial denaturation at 94°C for 5 min: 35 cycles of denaturation at 94°C for 30 s, annealing at 45°C for 30 s, extension at 72°C for 30 s, and final extension at 72°C for 5 min. PCR products were analyzed by 2% agarose gel. We also confirmed accuracy of AS-PCR via Sanger DNA sequencing.

### 2.3. Statistical Analysis

Hardy-Weinberg equilibrium (HWE) was tested by online HWE program (http://linkage.rockefeller.edu/ott/linkutil.htm) [28], and allele and genotype frequencies were examined by Fisher's exact (two-tailed) test, using SPSS version 10.0 software (SPSS for Windows, Inc., Chicago, IL). Odds ratios with 95% confidence intervals were derived for external variables, with *P* value <0.05 constituting statistical significance. Correction for multiple comparisons used positive false discovery rate (FDR) statistics based on the method of Benjamini and Hochberg [29], with FDR < 0.05 for significant association adopted as true positive result. Power was estimated by free power analysis program, G∗power 3.1 [30].

## 3. Results

Two SNPs of* ADIPOQ* and two SNPs of* TCF7L2* genes were genotyped in T2DM patients and controls, using allele-specific polymerase chain reaction analysis (Table 3). Distributions of* ADIPOQ* and* TCF7L2* genotypes are plotted in Table 4, and genotype frequencies were evaluated by chi-square test. The method of Benjamini and Hochberg (threshold = 0.1) served to correct for multiple testing of each tested gene. Results showed significant difference (*P* = 0.042) in* ADIPOQ* (rs1501299 (G276T)) polymorphism after Benjamini and Hochberg correction (FDR < 0.05). Yet no differences of genotype frequencies in* ADIPOQ* (rs2241766 (T45G)) and* TCF7L2* (rs7903146) polymorphisms were detected. Surprisingly, genotypes of rs12255372 for* TCF7L2* were the only GG genotype appearing in both healthy and T2DM subjects: that is, rs12255372 is not informative among the Taiwanese population. Genotype distributions of all SNPs do not deviate from HWE in either T2DM patients or controls (data not shown).

Similar results were observed in allele frequency: minor allele frequency of rs1501299 SNP in* ADIPOQ* being 28.5% higher among T2DM patients compared to healthy controls (20.6%, *P* = 0.028, FDR < 0.05) (Table 5). The odds ratio (OR) showed T allele of rs1501299 SNP as the risk factor for T2DM in our subjects (OR = 0.59, 95% confidence interval (CI): 0.01–0.24, *P* = 0.032), with no difference apparent in other polymorphisms. Collectively, these results suggest genetic variation of* ADIPOQ,* in particular rs1501299 SNP, may have a role in prevalence of T2DM among the Taiwanese population. We likewise compared distribution of genotypes for* ADIPOQ* rs1501299 SNP among Taiwanese T2DM and control subjects from the current study, plus other ethnic control groups: for example, the European (CEU), the Han Chinese (HCB), and the Nigerian (YRI) groups from HapMap database (Table 6). While GG genotype in rs1501299 had prevalence in all control ethnic groups, TT genotype is relatively higher in Taiwanese T2DM patients, such that T allele of rs1501299 in* ADIPOQ* might play an important role in T2DM.

## 4. Discussion

Novel associated genetic diseases identified by genome-wide association studies are needed for further testing in different populations. Association study is the most important tool for identifying genes conferring susceptibility to complex disorders. Genetic studies on many complex disorders like diabetes and psychiatric traits confirm many risk-associated genetic variants contributing to a subtle effect [2]. Our case-control study analyzed association of T2DM with* ADIPOQ* and* TCF7L2* polymorphism in the Taiwanese. Interestingly, significant correlation was noted between* ADIPOQ* rs1501299 polymorphism and T2DM subjects, with T allele shown as a risk factor for prevalence of T2DM.

*Polymorphisms of TCF7L2. TCF7L2* is involved in beta-catenin-dependent Wnt signaling pathway, which employs its physiological functions in pancreatic and intestinal endocrine cells [20, 21]. Previous investigations correlated* TCF7L2* with risk of T2DM in extensive populations [31, 32], though not including the Taiwanese. We rated association of* TCF7L2* polymorphisms rs7903146 and rs12255372 with T2DM prevalence in Taiwan. However, minor allele frequencies of rs7903146 and rs12255372 are 0.023 and unknown, inconsistent with prior study of the Chinese population [33]. Data hinted these SNPs are not polymorphic in our population and were removed from the final analyses. Although polymorphisms of* TCF7L2* were rare in Taiwanese population, it is noteworthy to observe linkage between rs7903146 in* TCF7L2* and T2DM in ethnic groups [31, 34–37]. Genetic markers well represented in one population but showing significantly different allele frequencies, even almost total absence in another allele across ethnicities, are frequently observed [38]. These phenomena might result from heterogeneity among ethnic groups; their effects on susceptibility to T2DM are unclear and merit follow-up study.

*Polymorphism of ADIPOQ*. Adiponectin, adipocytokine encoded by* ADIPOQ*, is central to insulin sensitivity and resistance and other metabolic syndrome traits in diverse populations [39, 40]. In addition, plasma level of adiponectin was proposed as a biomarker for T2DM, hypertension, and obesity [41, 42];* ADIPOQ* SNPs were linked with plasma adiponectin level among the Caucasian population [11, 12] and metabolic syndrome [9, 10].* ADIPOQ* polymorphisms and T2DM or diabetic nephropathy had also been evaluated from many populations: for example, the European-derived [17, 18], the Asian [14, 16, 40], the non-European South African [43], and the African American [44] populations. Still, no difference appeared between these SNPs and T2DM in the Taiwanese population [45]. Gao et al. indicated G allele frequency of* ADIPOQ* SNP (rs2241766) as prevalent in metabolic syndrome patients versus control groups in the Chinese Han population [10], which demonstrably augments risk of T2DM in the Chinese population [10]. We analyzed genetic association between* ADIPOQ* polymorphisms and T2DM in 149 patients and 139 normal controls in Taiwan (study power = 0.7); genotype frequency of SNP rs2241766 showed no difference between T2DM and controls. Since rs2241766 is a silent mutation for Gly15, this SNP may link with other functional SNPs.

We focused on other SNPs of* ADIPOQ*, rs1501299, to find rs1501299 SNP definitely associated with T2DM (*P* = 0.045). High prevalence of T allele (*P* = 0.028) and risk of T2DM in the Taiwanese population was observed (*P* = 0.032), findings that are consistent with studies performed in populations of southern India [14], Japan [15, 16], and Mainland China [46]. Also, Yang et al. reported SNP rs1501299 linked with obesity, metabolic syndrome, and diabetes in the elderly Taiwanese [47], which suggest that T allele of SNP rs1501299 increases risk of diabetes among the Chinese population, consistent with our data. However, these results conflicted with other studies of Chinese people [48–51], suggesting that SNP rs1501299 may not be a functional SNP but most likely be in linkage disequilibrium with other functional variants [47]. Moreover, it suggests genetic background of the Taiwanese may be unique compared to the Mainland Chinese. Prevalence of TT genotype in SNP rs1501299 noted among Taiwanese was compared with controls from Europe, China, and Nigeria in HapMap database. Results indicate most groups (Taiwanese, European, Japanese, and Nigerian) retain high levels of GG content, albeit not Mainland China (Table 6), suggesting genetic background of Chinese people is heterogeneous [46]. This portends a greater number of subjects from different populations to evaluate in the future.

*Study Limitation*. One limitation of this study was small sample size due to cost considerations. While genetic analysis of SNP rs1501299 proved significant, we could not entirely exclude a possibility of false positives because of sampling limitations and other factors (confounders with other medical conditions, subject assessment lacking support by structured interviews). Yet we added statistics, FDR < 0.05, as a true positive association to support our conclusions.

## 5. Conclusions

Our case-control study linked T2DM with* ADIPOQ* and* TCF7L2* polymorphism in Taiwan's Han Chinese population: strong correlation emerged between* ADIPOQ* rs1501299 and T2DM subjects in Taiwan. Taken together, this verifies that* ADIPOQ* is associated with T2DM risk in Taiwanese population, which may prove useful clinically as a genetic marker. Results suggest* ADIPOQ* as a key genetic contributor to T2DM that may serve as a clinical biomarker.

## Figures and Tables

**Table 1:** Characteristics of participants.

	T2DM (*n* = 149)	Controls (*n* = 139)
Age (years)	65.62 ± 13.48	27.08 ± 14.58
Sex: male/female	77/72	93/44

Values represented mean ± SD (standard deviation).

**Table 2:** Primers utilized in allele-specific PCR analysis.

Gene/SNP	Primers, 5′-3′	Product size
*ADIPOQ/*rs2241766	F: GCAGCTCCTAGCCGTAGACTCTGCTG R: GGAGGTCTGTGATGAAAGAGGCC FG: TGCTATTAGCTCTGCCCGAG FT: TGCTATTAGCTCTGCCCGAT	372 bp 226 bp

*ADIPOQ/*rs1501299	F: CCTGGTGAGAAGGGTGAGAA R: AGATGCAGCAAAGCCAAAGT FG: TGATATAAACTATATGAATG FT: TGATATAAACTATATGAATT	241 bp 168 bp

*TCF7L2/*rs7903146	F: GCCTCAAAACCTAGCACAGC R: GTGAAGTGCCCAAGCTTCTC FC: GCTAAGCACTTTTTAGATGC FT: GCTAAGCACTTTTTAGATGT	220 bp 176 bp

*TCF7L2/*rs12255372	F: CTTGAGGTGTACTGGAAACTAAGGC R: CTGTCTATTTGGCATTCAAATGGA FG: GGAATATCCAGGCAAGAACG FT: GGAATATCCAGGCAAGAACT	251 bp 130 bp

SNP: single nucleotide polymorphism; F: forward; R: reverse.

**Table 3:** Polymorphisms genotyped in this study.

Marker type	dbSNP ID	Position in genes	Alleles	
			DNA (major/miner)	Amino acid
*ADIPOQ *				
SNP	rs2241766	Coding sequence	T/G	NAC
SNP	rs1501299	Intron 2	G/T	NAC

*TCF7L2 *				
SNP	rs7903146	Intron 3	C/T	NAC
SNP	rs12255372	Intron 4	G/T	NAC

NAC: no amino acid change.

**Table 4:** Genotype frequencies (%) of SNPs: T2DM and controls.

dbSNP ID	*n*	Genotypes (%)			*P* value	Dominant		Recessive	
						OR (95% CI)	*P* value	OR (95% CI)	*P* value
*ADIPOQ*		TT	TG	GG		TG + GG/TT		TT + TG/GG	

rs2241766									
T2DM	149	71 (47.7)	69 (46.3)	9 (6)	0.967	0.91 (−0.10~0.14)	0.684	1.49 (−0.18~0.07)	0.39
Control	139	70 (50.4)	57 (41)	12 (8.6)					

*ADIPOQ *		GG	GT	TT		GT + TT/GG		GG + GT/TT	

rs1501299									
T2DM	149	80 (53.7)	53 (35.6)	16 (10.7)	0.042∗	0.59 (0.01~0.24)	0.032∗	0.66 (−0.07~0.2)	0.32
Control	139	92 (66.2)	37 (26.6)	10 (7.2)					

*TCF7L2 *		CC	CT	TT				CC/CT	

rs7903146									
T2DM	149	143 (96)	6 (4)	0 (0)	0.246			1.85 (−0.09~0.22)	0.25
Control	139	129 (92.8)	10 (7.2)	0 (0)					

*TCF7L2 *		GG	GT	GG					

rs12255372									
T2DM	149	149 (100)	0 (0)	0 (0)	NA				
Control	139	139 (100)	0 (0)	0 (0)					

*P < 0.05; NA: nonavailable; OR: odds ratios; CI: confidence interval.

**Table 5:** Allele frequencies (%) of SNPs *ADIPOQ* and *TCF7L2*: T2DM and controls.

SNP types	*n*	Allele (%)		*P* value	OR (95% CI)
*ADIPOQ*		T	G		

rs2241766					
T2DM	149	211 (70.8)	87 (29.2)	0.968	1 (−0.08~0.08)
Control	139	197 (70.7)	81 (29.3)		

*ADIPOQ*		G	T		

rs1501299					
T2DM	149	213 (71.5)	85 (28.5)	0.028∗	0.65 (0.01~0.15)
Control	139	221 (79.4)	57 (20.6)		

*TCF7L2*		C	T		

rs7903146					
T2DM	149	292 (97.7)	6 (2.3)	0.252	1.82 (−0.04~0.01)
Control	139	268 (96.3)	10 (3.7)		

*P < 0.05; OR: odds ratios; CI: confidence interval.

**Table 6:** * ADIPOQ *SNP rs1501299 genotype distribution in this study and HapMap.

	*n*	Distribution of genotypes, *n* (%)		
		TT	TG	GG

Taiwanese				
T2DM	149	16 (10.7)	53 (35.6)	80 (53.7)
Control	139	10 (7.3)	36 (26.3)	91 (66.4)

		TT	TG	GG

HapMap populations				
HapMap-CEU∗	116	8 (6.9)	46 (39.7)	62 (53.4)
HapMap-HCB∗	90	8 (8.9)	42 (46.7)	40 (44.4)
HapMap-YRI∗	88	6 (6.8)	40 (45.5)	42 (47.7)

*CEU: Europe; HCB: China; YRI: Nigeria.
